# An Event-Classification Neural Network Approach for Rapid Railroad Bridge Impact Detection

**DOI:** 10.3390/s23063330

**Published:** 2023-03-22

**Authors:** Omobolaji Lawal, Shaik Althaf V. Shajihan, Kirill Mechitov, Billie F. Spencer

**Affiliations:** Department of Civil and Environmental Engineering, University of Illinois, 205 N. Matthews Ave, Urbana, IL 61801, USA

**Keywords:** impact detection, event classification, railroad bridge, wireless sensors, artificial neural networks

## Abstract

Railroads are a critical part of the United States’ transportation sector. Over 40 percent (by weight) of the nation’s freight is transported by rail, and according to the Bureau of Transportation statistics, railroads moved $186.5 billion of freight in 2021. A vital part of the freight network is railroad bridges, with a good number being low-clearance bridges that are prone to impacts from over-height vehicles; such impacts can cause damage to the bridge and lead to unwanted interruption in its usage. Therefore, the detection of impacts from over-height vehicles is critical for the safe operation and maintenance of railroad bridges. While some previous studies have been published regarding bridge impact detection, most approaches utilize more expensive wired sensors, as well as relying on simple threshold-based detection. The challenge is that the use of vibration thresholds may not accurately distinguish between impacts and other events, such as a common train crossing. In this paper, a machine learning approach is developed for accurate impact detection using event-triggered wireless sensors. The neural network is trained with key features which are extracted from event responses collected from two instrumented railroad bridges. The trained model classifies events as impacts, train crossings, or other events. An average classification accuracy of 98.67% is obtained from cross-validation, while the false positive rate is minimal. Finally, a framework for edge classification of events is also proposed and demonstrated using an edge device.

## 1. Introduction

**Figure 1:**
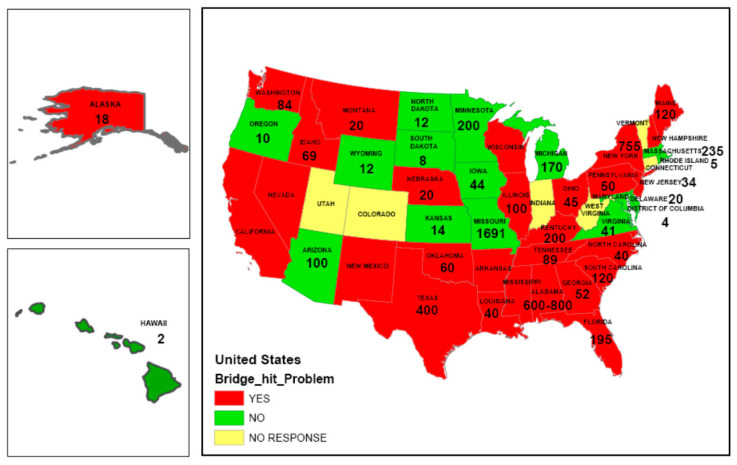
Number of bridge impacts between 2005 and 2008 across the United States [2].

Low-clearance bridges are susceptible to frequent impacts from over-height vehicles. Note that while major full-on impacts are relatively rare, minor impacts where vehicles scrape underneath bridges can occur frequently for such bridges. Figure 1 shows the results from a survey conducted by Agrawal et al. [1] detailing the number of highway and railroad bridge impacts from over-height vehicles that occurred between 2005 and 2008 across the United States. They also indicated that most state agencies consider bridge impacts to be a major problem.

Of the approximately 100,000 railroad bridges in the United States, a significant fraction are over 100 years old [2]. Some of these old bridges do not meet the current minimum vertical clearance standards, as they were designed before current requirements were imposed [3]. These low-clearance railroad bridges are therefore prone to impacts from over-height vehicles. For example, in the study of Agrawal et al. [1], 284 of the 826 recorded impacts on local bridges in New York were on railroad bridges. To further highlight the severity of this bridge impact issue, Joy et al. [4] investigated the leading causes of railroad bridge service disruptions between January 1999 and March 2010. They found that railroad bridge impacts from over-height vehicles on highways contributed to nearly half of these interruptions in service. As a result of these impact events, the railroad bridge structure may become damaged [5]. Moreover, aside from the damage to the railroad bridge infrastructure, impacts also result in a loss of revenue for rail authorities. Joy et al. [4] reported that a minor railroad bridge impact which causes negligible damage still results in an estimated loss of around $10,000, while a severe impact may cost up to $1.5 million for the bridge owner.

The above data shows that bridge impacts are a significant problem with huge cost implications for the managing authorities. Impacts to railroad bridges that go unnoticed can lead to its failure or result in derailments [6]. Impact events also have the potential to cause significant traffic delays, which can lead to substantial loss in revenue. Thus, in addition to assessing the condition of the structure after impacts, rapid impact detection allows the railroad bridge engineers to reduce the duration of traffic delays caused by the impact event. As a result, a system is needed that can quickly and accurately detect the occurrence of railroad bridge impacts to ensure reliable operation, as well as to minimize losses due to downtime.

While several studies have been carried out on impact detection for aerospace structures [7,8,9,10,11], few researchers have developed systems capable of detecting impacts for both highway and railroad bridges. Some examples include Song et al. [12], who used piezoelectric sensors embedded in a concrete block utilized for representing a highway bridge girder to detect impacts. The impacts were simulated using a truck model in the laboratory and are detected once the predetermined threshold is exceeded. Furthermore, a camera is triggered upon detection of the impact to validate the occurrence of the event. Byrne [13] reported that the Mass Transit Rail in Hong Kong used fiber optic sensors to develop a ship collision notification system to report when the railroad bridge over the Rambler Channel has been struck by a ship. Yang [14] developed a system for localizing impacts on bridges in cold remote regions using strain sensors and an inverse algorithm. Sun et al. [15] demonstrated how structural health monitoring could be employed for detecting ship collisions with highway bridges. The study monitored the displacements and triggered an alarm once the set threshold was exceeded, allowing engineers to carry out visual inspection of the bridge. Fu et al. [16] recently developed a wireless monitoring system for rapid condition of long-span bridges subjected to impacts. However, all of these strategies involve the use of predetermined thresholds to detect impacts. In the case of railroad bridges, the use of such vibration thresholds for impact detection is prone to misclassification of events, due primarily to the fact that train crossings can also generate significant responses. For example, wheel flats, which may result from a train’s axle locking up during braking, can cause extremely high acceleration responses in railroad bridges [17].

Some researchers have proposed machine learning to classify railroad bridge events and detect impacts [18,19]. For example, Sitton et al. [18] proposed a machine learning approach for impact detection, where a simple neural network was used to differentiate between train crossings and impact events on railroad bridges. The approach was further developed by using an ensemble of neural networks for classification to improve the accuracy from the previous work [19]. However, the training datasets are heavily imbalanced, with most of the data coming from train crossings rather than impacts. This imbalance makes the trained models less robust. While the use of an ensemble of neural networks may improve the accuracy of event classification, training time can be excessive, which may also lead to models requiring excessive amounts of memory to execute [20,21]. Moreover, these systems are based on traditional wired sensor deployments and central data collection and analysis. Recently, researchers (e.g., Ozdagli et al. [22], Xu et al. [23]) carried out experimental investigations on the use of crash beams to attenuate damage to railroad bridge girders due to impacts from over-height vehicles. To date, an accurate impact detection system for railroad bridges that can enable rapid assessment is not available. Edge computing is particularly suitable for time-sensitive applications such as bridge impact detection [24]. Edge computing deploys computation resources at the location where the data is acquired (i.e., data does not have to be collected at a centralized hub). Thus, the use of edge computing for classifying railroad bridge events and subsequently detecting impacts may prove to be beneficial and aid the rapid reporting of impacts to engineers.

In this paper, an approach for rapid impact detection for railroad bridges that can be deployed on the edge of smart wireless sensors is proposed. To provide a robust assessment, the proposed approach uses an event classification algorithm to first determine whether an event is a freight train crossing, because this type of event is the most common and also of interest to railroad bridge engineers. If an event is not identified as a freight train crossing, the algorithm uses a neural network classifier to determine between passenger train crossings and minor impact events which produce responses of the same order of magnitude; for these cases, a simple threshold approach for classification is not effective. The classification procedure involves extracting key features from event-triggered wireless sensing data to train a neural network that is sufficiently lightweight to be deployed directly on an edge device. The trained neural network model is then evaluated against test data and shown to be effective at classifying events as either impacts, train crossings, or other events. The paper is organized as follows: First, Section 2 discusses the challenges with using wireless monitoring systems for impact detection. Section 3 describes the proposed event classification neural network, as well as the associated training. Section 4 presents the efficacy of the proposed approach using a test dataset. A framework for future implementation of the proposed system on edge devices is also presented. Finally, Section 5 concludes the paper. The main contribution of this work is the development of an efficient and accurate approach for rapid detection on railroad bridge impacts that can be implemented at the edge on a state-of-the-art wireless sensor. The results demonstrate the accuracy and efficiency of the proposed model.

## 2. Challenges of Using Wireless Monitoring Systems for Impact Detection

Certain challenges exist when deploying wireless monitoring systems for railroad bridge impact detection. A major issue is that wireless smart sensors are mainly powered by batteries, which have limited capacity. As a result, most wireless sensing solutions deployed for structural health monitoring tasks employ duty cycling to prevent depletion of the power source [25]. Researchers have also proposed several methods for minimizing the power consumption of wireless sensors, including balancing energy consumption among nodes considering transmission distance to the base station [26], energy-efficient communication protocols [27,28], and energy-efficient routing algorithms [29,30]. However, bridge impacts are sudden events which can be missed if periodic monitoring is utilized, because they can occur while the sensors are in sleep mode. Therefore, to capture sudden events, wireless sensors must always be powered on. The above solutions are ineffective for continuous monitoring. Furthermore, wireless sensing platforms are typically comprised of microprocessors with limited computational capabilities and memory. Therefore, both limited power and available memory play a fundamental role in developing solutions for railroad bridge impact detection using wireless monitoring systems.

**Figure 2:**
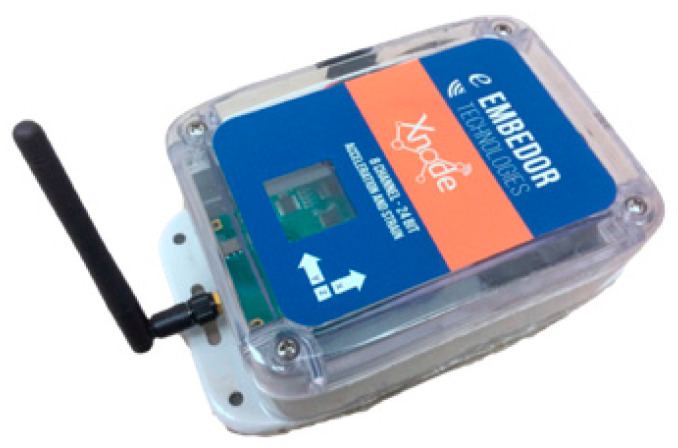
Enclosed Xnode smart sensor.

To address the issue of power consumption while monitoring sudden events, Fu et al. [31] proposed a solution that utilizes an ultralow power trigger accelerometer, the ADXL362 from Analog Devices, combined with the Xnode [32,33,34,35] to enable continuous monitoring; the Xnode is a wireless smart sensor (see Figure 2) platform that has been developed and demonstrated to be effective for structural health monitoring of civil engineering infrastructure. Due to its low power consumption, the ADXL362 can be powered continuously to monitor structural vibrations [36], while the primary wireless sensor hardware is sleeping in a lower-power mode. When the vibration level exceeds a predetermined threshold, the ADXL362 triggers an interrupt pin that wakes up the Xnode to carry out high-fidelity sensing using the ST Micro LIS344ALH. Once the vibration level falls below the threshold, another interrupt pin is set on the ADXL362 to notify the Xnode to return to deep sleep. This solution ensures that sudden events are not missed and is therefore employed in this work.

For the impact monitoring discussed in this paper, an event classification algorithm which helps to detect impacts is run on the data acquired from the Xnode (using the strategy discussed above). Implementation of the algorithm directly on the Xnode (i.e., at the edge) is desired. As such, the processing and memory requirements of the algorithm must be sufficiently lightweight to enable direct execution. An edge computing implementation on smart wireless sensors may also provide the additional benefit of reducing power consumption, as no internet connection is required [37]. Because the Xnode is the platform for which the algorithm is designed to be deployed, processing and memory requirements must be respected. For reference, the Xnode has a 204 MHz processor and 32 mb SDRAM. The development of the algorithm for impact detection is discussed in the next section.

## 3. Rapid Impact Identification Strategy

This section discusses the impact detection approach proposed in this paper. First, a comparison between typical impact and train crossing events is provided. Then, an event classification algorithm is proposed to address the problem of data imbalance that often hinders delineation between ambiguous railroad bridge events. Note that most of the data obtained corresponds to train crossing events (both freight and passenger trains). While distinguishing between freight train crossings and impact events is easy, the task is much more challenging for passenger train crossings or other events (e.g., HiRail vehicles). Therefore, a reliable heuristic method is first used for identifying freight train crossings and removing them from the dataset used for training the neural network. This approach also allows for a more balanced training dataset. The technique of removing examples from the majority class is a common approach for handling data imbalance [38]. Finally, the neural network architecture, the features used for training, as well as the training, testing, and validation approach are discussed.

### 3.1. Event Description

**Figure 3:**
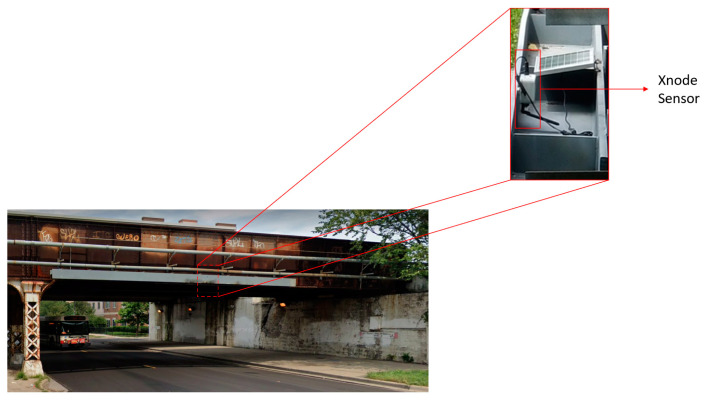
Low-clearance railroad bridge instrumented with the Xnode.

**Figure 4:**
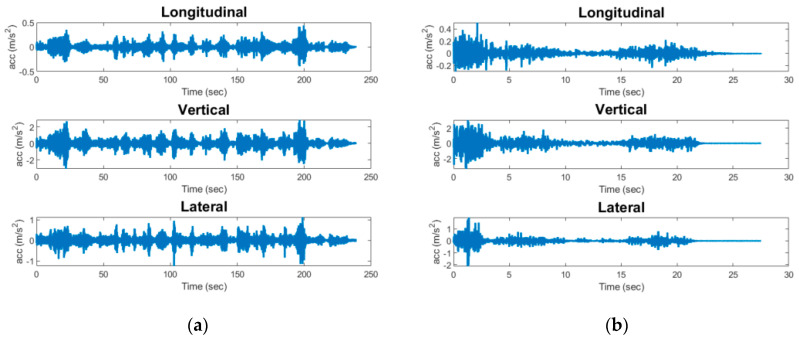
Sample data collected using the Xnode for a train crossing of a short rail bridge over a highway: (**a**) freight train; (**b**) passenger train.

**Figure 5:**
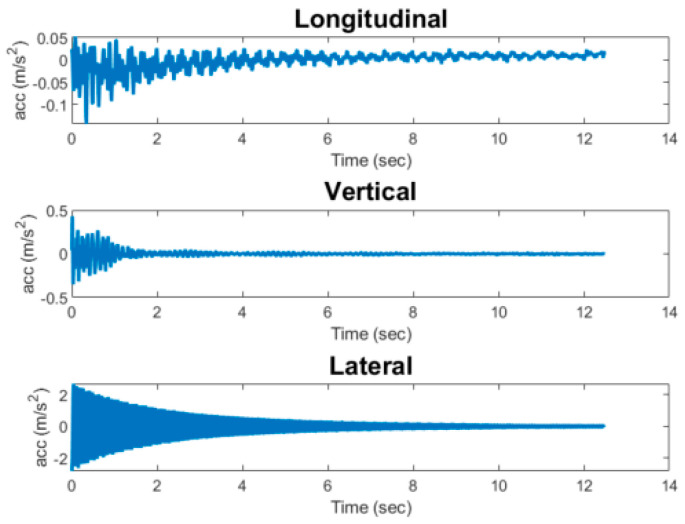
Data collected for a minor impact event using the Xnode.

While sensing data obtained from large impact events can be readily identified, data from minor impacts and train crossing events are often ambiguous. Thus, the three main categories of events are recorded by the Xnode sensor, i.e., (i) freight train crossings, (ii) passenger train crossings, and (iii) bridge impacts. For all events, a sampling rate of 100 Hz is used with the ADXL362 accelerometer, while the ST Micro LIS344ALH data are originally sampled at 1 KHz before being downsampled to 100 Hz. Figure 3 shows an instrumented railroad bridge in Chicago, IL, with the Xnode sensor attached to the crash beam and near the midspan of the bridge. Note that for short single-span bridges such as the one considered in this study, the responses obtained from the sensor are expected to be similar irrespective of its location and thus are able to capture the significant modes contributing to the response. Additionally, the crash beam for the bridge used in this study is mounted to the bridge deck. Figure 4 and Figure 5 show examples of train crossing events and a minor impact event on the bridge; these data were collected by the Xnode. As can be seen from the figures, peak accelerations due to train crossings are the same order of magnitude as the impact events; therefore, exceeding an acceleration threshold is not reliable for determining the occurrence of an impact. Moreover, as mentioned previously, the obtained dataset from the instrumented railroad bridges is limited and unbalanced, as over 99% of the data collected are from train crossings (both passenger and freight trains). While a neural network can be used directly to classify the different events, the features extracted from both freight and passenger trains crossings tend to be similar; therefore, from the preliminary analysis carried out, the use of a neural network classifier was found to be prone to misclassification of both events. The above factors highlight the need to have a more robust approach for classification of railroad bridge events. To address these problems, an event classification algorithm is proposed to classify events that occur on railroad bridges as impacts, train crossings, or other events. Furthermore, freight trains are identified to give additional information that enables engineers carry out further analysis on the response of railroad bridges to train crossings. This identification is carried out based on the event duration as is shown in the next subsection.

### 3.2. Proposed Event Classification Algorithm Approach

**Figure 6:**
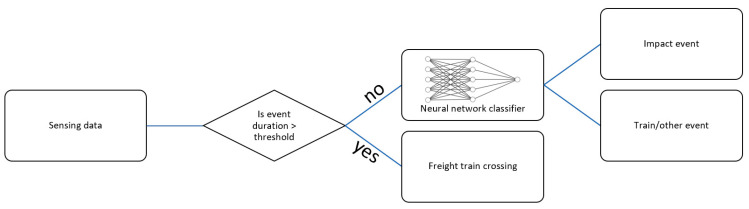
Algorithm for event classification.

This paper proposes an approach to classify events on railroad bridges. First, the underlying patterns in the different event data are studied. The events lasting longer than 30 s were noted to be exclusively due to freight train crossings. Therefore, the first check in this approach is with respect to the duration of an event. Note that an event is only recorded if the vibration exceeds a certain predetermined threshold in the ADXL362 accelerometer. If its duration is greater than 30 s, then the event will be automatically classified as a freight train without the need for further analysis. Note that this event duration threshold can be generalized due to the known typical length of freight trains. However, for events shorter than 30 s, further analysis is still needed. A neural network classifier is employed to classify such events as impact, train crossings, or other events. The event classification algorithm is shown in Figure 6.

### 3.3. Neural Network Classifier

A railroad bridge’s response to dynamic loads such as train crossings and impacts is dependent on many factors. Examples of these factors are bridge characteristics including its span length, mass, stiffness, damping, vehicle speed and weight, etc. [39]. The relationship between these factors and bridge response is complex and nonlinear. Therefore, in this work, an artificial neural network (ANN) is employed to learn the underlying complex relationships between features extracted from railroad bridge responses and the events producing the response. The purpose of utilizing an ANN is to facilitate the accurate classification of events which occur on railroad bridges. The ANN can learn such complex relations because of the use of activation functions.

#### 3.3.1. Neural Network Architecture

**Figure 7:**
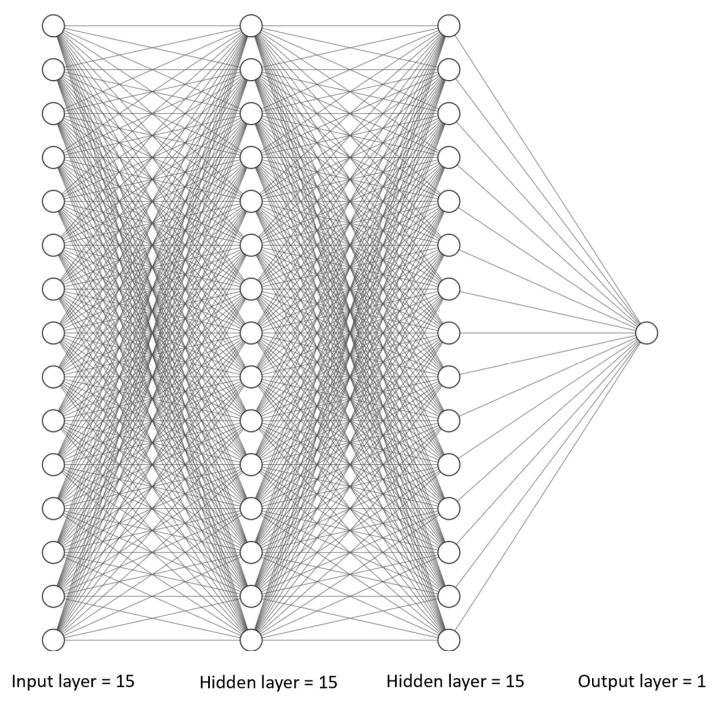
Fully connected neural network architecture for railroad bridge event classification.

After an iterative process, which entailed experimenting with various ANN architectures, a simple and efficient architecture which optimized the ANN performance was chosen. A fully connected neural network is implemented. The network consists of two hidden layers, with each layer comprising fifteen nodes, as illustrated in Figure 7. Furthermore, the rectified linear unit (ReLU) activation function was applied to each hidden layer. ReLU is a piecewise linear function that outputs the input directly for a positive input and otherwise outputs zero. This ReLU function is a commonly used activation layer in neural networks because its use makes models easier to train and generally leads to better performance [40]. The sigmoid activation function was used for the output layer. The reason for employing a sigmoid activation function on the output layer is to ensure the output is always a probability, i.e., a number between 0 and 1 [41]. The sigmoid function is desirable for binary classification problems. In this study, the two categories of interest are minor impact events and train crossing/other events. Note that all the impact data used in this work are minor impact events. A classification threshold of 0.5 is employed to distinguish between the two classes. The ANN was established using the Keras Sequential Applications Program Interface in Python.

#### 3.3.2. Feature Extraction

To use the ANN architecture described above, a set of inputs needs to be fed into the network. These inputs are features extracted from the acceleration response from both impact and train crossing events on railroad bridges. The features chosen in this work build on the prior investigation of Sitton et al. [18,19]. The carefully selected features are (i) maximum absolute acceleration from Xnode’s high-fidelity data acquisition; (ii) the two most dominant frequencies in the Fast Fourier Transform (FFT) of the response; (iii) center of mass and (iv) spectral energy. Each of the five features is extracted from the three acceleration directions, making a total of fifteen inputs into the ANN. These features can help differentiate between impact and train crossing events on railroad bridges.

The data acquired from the Xnode are the acceleration response in three directions, namely the vertical, longitudinal, and lateral. While train responses may not produce significant responses in the longitudinal direction, impact events sometimes generate significant vibrations in all three directions. Therefore, acceleration responses from all three directions are used as input into the ANN to help distinguish between train crossings and impact events. For feature extraction, first, the maximum absolute acceleration is extracted from responses in all directions. This computation is done for both the ST Micro LIS344ALH sensing data and the ADXL362 data. To ensure the use of a zero-mean signal, the mean is subtracted from the obtained acceleration signals before calculating the maximum absolute value in each of the coordinate directions. The ADXL362 uses a FIFO buffer, and the data are recorded from a few moments before an event to about 2 s after the event occurs, allowing the Xnode to boot and begin collecting data about 1 s after the start of the event [31]. Using the ADXL362 acceleration data in combination with the high-fidelity acceleration data from the ST Micro LIS344ALH offers the unique capability of capturing all data points during the event and thus ensuring that the largest acceleration for an entire event is obtained. The reason for using both ADXL362 and ST Micro LIS344ALH is that sometimes the maximum acceleration occurs before the ST Micro LIS344ALH sensing takes over, while at other times it occurs afterward.

Next, the frequency domain response is also used as a feature by taking the FFT of the high-fidelity acceleration in each direction. The dominant frequencies for impact events are expected to differ from those for train crossing events. This difference is because an impact induces free vibration of the bridge, which would be dominated by the natural frequencies of the bridge or the structural element to which the sensors are attached, whereas a train crossing produces a forced vibration which will include the forcing frequencies in addition to the natural frequencies of the bridge. Note that while the dominant frequencies between passenger trains and major full-on impacts are expected to be clearly different, the difference is more ambiguous in the case of minor impacts which frequently occur on railroad bridges. Thus, the use of dominant frequencies as the sole feature for identifying impacts may not produce highly accurate results. In this paper, the frequencies with the two highest magnitudes from each direction are used as input to the neural network. Note that the Xnode has a five-second inactivity detection period; therefore, all event records will have to last a minimum of five seconds. Therefore, the number of recorded data points will be sufficient to obtain the dominant frequencies after taking the FFT.

Furthermore, Sitton et al. [18] define a term, denoted by the normalized center of mass of the signal to delineate between events. The normalized center of mass is simply the centroid of the signal in time domain and is given by

(1) $$\bar{T}=\frac{1}{t_{N}}\left(\frac{\sum_{i=1}^{N}\left|{\ddot{x}}_{i}\left(t_{i}\right)\right|t_{i}}{\sum_{i=1}^{N}\left|{\ddot{x}}_{i}\left(t_{i}\right)\right|}\right)$$

where $\bar{T}$, N, $\ddot{x}$, and t are the normalized center of mass, total number of data points, ADXL acceleration for a particular direction, and time, respectively. The normalized center of mass will result in a number between 0 and 1. For impact events, the center of mass is expected to be towards the beginning of the signal, while for train crossing events the responses are expected to have a normalized center of mass near 0.5.

Finally, the spectral energy of the high-fidelity acceleration in each direction is also computed and used as input to the ANN. As mentioned earlier, the impact event typically induces a free vibration, while train crossing events are forced vibrations. As a result, train crossing event responses are expected to contain more frequencies than impact event responses. Therefore, the spectral energy from train crossing events is expected to be greater than that from impacts. In this paper, the spectral energy is computed by taking the sum of the power spectral density (PSD) magnitudes obtained from the high-fidelity acceleration in all directions across all frequencies. In total, fifteen inputs are fed into the ANN model created in Section 3.3.1. Table 1 shows a summary of the features extracted as inputs to the ANN.

#### 3.3.3. Training and Testing Approach

The training and testing dataset used in this study was obtained by extracting the aforementioned features from sensor data. These data were acquired by instrumenting two railroad bridges with the Xnode. To ensure the trained model is robust, the number of train crossings and impact events in the dataset used for creating the ANN model are equal. The dataset contains a total of 210 events with train crossings and minor impacts having 105 events each. Note that major impacts are quite infrequent, therefore getting a large dataset for such events is difficult. This dataset is randomly split into 70% training data and 30% testing data. Furthermore, 20% of the data split for training was held out as a validation dataset used during the training and model development process. The test data were only used after the full model had been developed.

Standardization of training features is a common approach in many machine learning problems. This approach is usually implemented to ensure the model learns all features correctly, even if a feature has a greater order of magnitude compared to the others [42]. In this paper, the standardization and preprocessing are done before training the ANN model by removing the mean and scaling to a unit variance. The standardization is only carried out for the training data, and the obtained parameters are then used to transform the test data. For training, the loss function minimized is binary cross-entropy. This function is used for binary classification applications and computes the cross-entropy between actual and predicted labels. The Adam Optimizer is employed to update the parameters to minimize the loss. However, note that some researchers have recently adopted metaheuristic evolutionary algorithms for the optimization of neural networks [43,44]. Other model parameters used are a learning rate of 0.001, which generally works well in most cases, and an epoch size of 200 was chosen after a trial-and-error approach. A detailed description of these parameters can be found in the Keras documentation [45]. Finally, during the training, some of the data are held out and used for validation. Table 2 gives a summary of the hyperparameters used in training the ANN model.

Having described the approach for training the ANN model, the results obtained from testing are discussed in the next section.

## 4. Results and Discussion

In this section, the accuracy of the developed ANN model is validated through evaluation of the test data. The accuracy is verified by comparing the model predictions with the actual labels. To further ensure the robustness of the model, a cross-validation approach is also used. Finally, a framework for edge impact detection is proposed.

### 4.1. Neural Network Classifier Results

**Figure 8:**
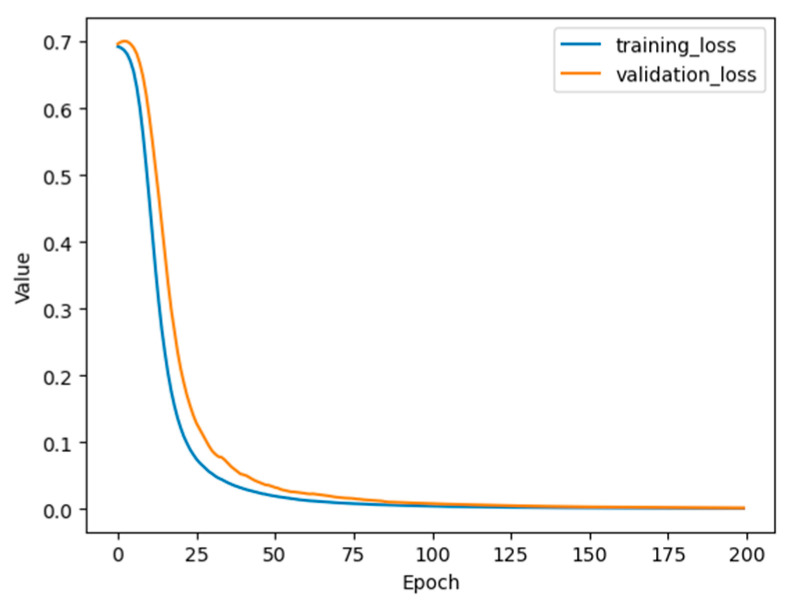
Training and validation loss curves for the ANN model.

**Figure 9:**
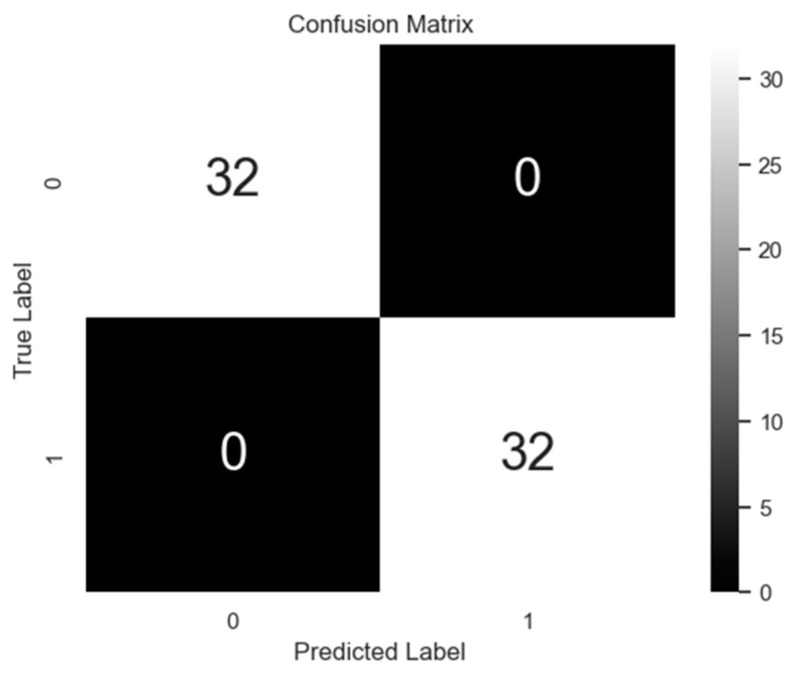
Confusion matrix of actual vs. predicted labels (0 = train crossing/other, 1 = minor impact).

Figure 8 shows the training loss curve of the ANN model described in Section 3, which demonstrates its training performance. Both training and validation losses decrease as the number of epochs increase and converge to a very low value. This reduction in the losses illustrates that the number of epochs used was adequate and that the model has the capability to perform well after training. Figure 9 shows the performance of the model on the test data through the confusion matrix. The ANN classifier achieved perfect accuracy on the held-out test data.

To further ascertain the robustness of the model, its performance was verified on multiple training instances using the *k*-fold cross-validation technique. Cross-validation is a commonly applied method to evaluate the accuracy of a machine learning model. The cross-validation process involves dividing the data into *k* groups. One group is held out for testing, while the remaining are used for training. This procedure is repeated *k* times. The performance of the model on each group’s data is used to validate its robustness [46]. In this paper, a 10-fold cross-validation procedure was applied, i.e., the dataset was split into 10 groups. The mean accuracy score obtained from the 10 different models was 98.67%, while the standard deviation was 0.0267. The mean accuracy from the cross-validation models was similar to that achieved by the model on the held-out test data shown above. Additionally, the low standard deviation shows that the model is not overfitted.

Moreover, other metrics were also used in evaluating the performance of the model during the cross-validation process. First, the area under the curve (AUC) of the receiver operating characteristic (ROC) curve was computed. The ROC curve is a plot of the false positive rate versus the true positive rate for different decision threshold values. The AUC score indicates the skill of a model. An average AUC score of 0.99 was obtained from the cross-validation process, showing that the model is very skillful, i.e., the model is highly likely to positively identify impact events. Other useful metrics to calculate include the precision and recall values. For this problem, the precision and recall give an indication of how good the model is at identifying impacts as well as the false positive rate. The precision is the ratio of actual impacts from all predicted impacts to the total number of predicted impacts. Thus, a high precision value implies a low false positive rate. On the other hand, recall is a measure of the proportion of impact events the model is able to identify. The F1 score, which is the harmonic average of the recall and precision was computed during the cross-validation process. The obtained average F1 score of 0.98 shows the model has a low false positive rate as well as capability of not missing impact events when they occur. These scores show the potential of deploying the model in practical applications. Table 3 gives a summary of the results obtained from the cross-validation process.

### 4.2. Discussion

This subsection shows the proposed framework for on edge railroad bridge event classification. The implementation of the developed ANN model on an edge device and its computational efficiency are discussed herein.

#### 4.2.1. Edge Classification Framework

Machine learning and structural health monitoring applications may have high computational requirements. The common approach to meet these computational needs is with cloud computing, where data are moved to a centralized location for processing. However, this approach can be challenging for applications that require real-time or near real-time inferences. These problems exist because cloud computing can take extra time in processing by sending data to a centralized location and potentially waiting in queue for previously uploaded data to finish processing. Furthermore, there could be a delay in uploading data due to connection issues. As the use of the internet-of-things (IoT) sensors to generate data for applications that require real-time decision-making becomes increasingly more popular, the need to have computational resources that can handle such demands becomes more urgent. Edge computing has been proposed by researchers as a feasible solution to this problem [47]. As impact detection requires the quick reporting of events to engineers, the potential of performing this task on edge can be hugely beneficial.

**Figure 10:**
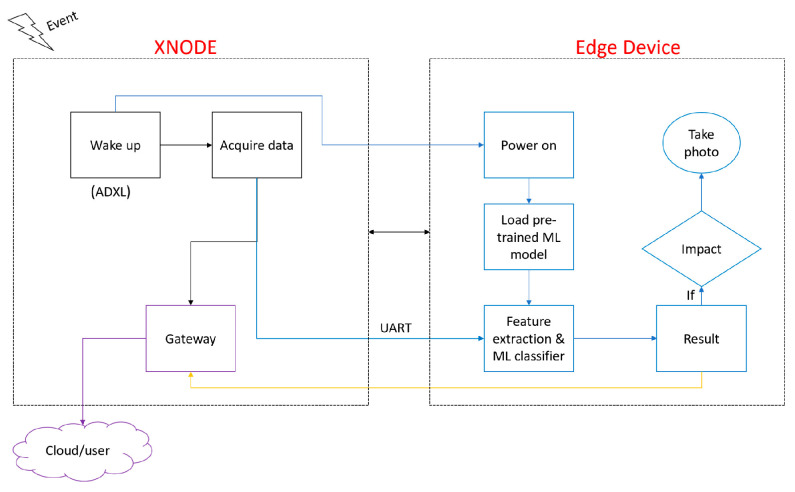
Framework for edge classification.

This work proposes a framework that will leverage the integration of an edge device with the Xnode platform. Upon the occurrence of an event, the ADXL362 trigger accelerometer wakes up the Xnode to start sensing. Simultaneously, the edge device will be powered on. Also, after power is turned on, the edge device loads the pre-trained neural network classifier described in Section 3. After the data acquisition task is completed, the Xnode communicates with the edge device and sends the acquired data using the UART interface. On the edge device, the acquired data are then passed into the ML classifier to classify the event. Next, the result obtained will be sent to the Xnode gateway sensor node. The gateway node then uploads the result to the cloud, and the end user is also notified if an impact event occurred. Finally, if the event is classified as impact, the edge device triggers its camera sensor to take a photo for validation. The proposed framework is illustrated in Figure 10. To demonstrate the feasibility of the proposed approach, the deployment of the developed ANN model on the Raspberry Pi microcontroller is discussed in the next section. The Raspberry Pi is chosen for the prototyping because the Xnode does not currently include machine learning libraries that would allow for the implementation of the developed model. However, note that once out of the research prototype phase, the end goal is to deploy the model on the Xnode by leveraging the study of V. Shajihan et al. [48]. The work developed a wireless smart vision sensor by integrating a low-cost and low power consumption machine vision camera with the Xnode sensor. This integration allows the Xnode to trigger the camera to take snapshots. Therefore, this study will allow activation of the camera system when the ANN model detects the occurrence of an impact. These pictures will be useful for confirming the occurrence of impacts as well as showing the offending vehicles, which may allow authorities to locate the drivers who may leave the scene.

#### 4.2.2. Implementation on Edge Device

At present, the Xnode software stack does not include the machine learning libraries needed to implement the neural network classifier (Keras, Tensorflow, etc.). Porting the necessary software to the Xnode is a substantial development task. For the purposes of rapid prototyping and evaluation of the proposed ANN method, instead we chose to employ a substitute platform with even lower memory capacity and computational capabilities than the Xnode that already meets these requirements. If the model can be used with satisfactory performance on this device, it can then be expected to perform no worse on the Xnode once the required libraries have been ported. The Raspberry Pi was selected as the evaluation platform. Thus, this section discusses the deployment of the ANN model on the edge of a Raspberry Pi, detailing the necessary steps to deploy the pretrained model on the microprocessor board.

**Figure 11:**
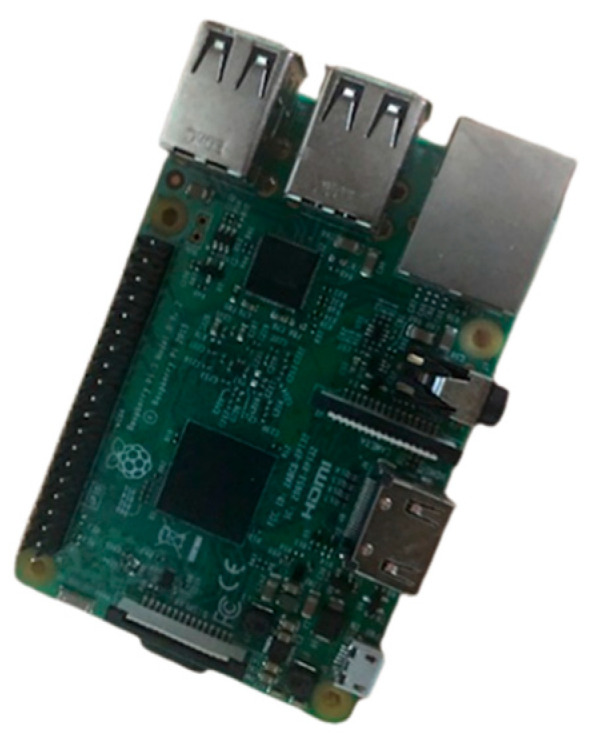
Raspberry Pi 3 Model B board.

The Raspberry Pi is an affordable small single board computer that can be used with a display screen like the typical computer. The computer also has built-in programming languages like Python, which allows users to develop custom scripts for their applications [49]. Raspberry Pi OS (previously called Raspbian) is the officially supported operating system for this computer [50]. The Raspberry Pi 3 Model B board, which is the third generation of Raspberry Pi, is used in this study. This model succeeds the original Raspberry Pi Model B+ and Raspberry Pi 2 Model B. Furthermore, the improvements include a faster processor and more effective wireless connectivity [51]. Figure 11 shows the Raspberry Pi 3 Model B board.

**Figure 12:**
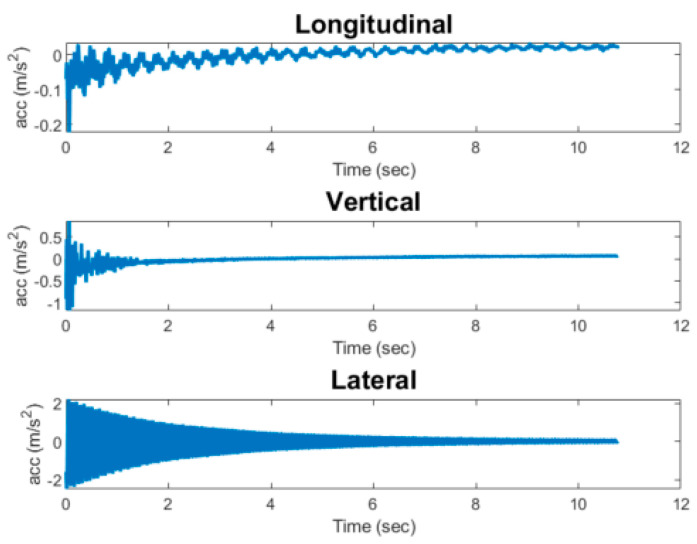
Acceleration record of test event.

The original Keras model developed is lightweight enough to be deployed on the Raspberry Pi device, as its memory size is 36 KB. However, for optimized performance on an edge device, the model was converted into a TensorFlow Lite format. The generated model is 4 KB in size. TensorFlow Lite is designed to enable users make predictions on edge devices [52]. The model conversion from a high-level Keras model to TensorFlow Lite was done using the TensorFlow Lite Python API. The model was then loaded into the Raspberry Pi memory after generating the TensorFlow Lite version. Next, the impact detection algorithm was deployed on the Raspberry Pi. To test the aforementioned implementation, the data showed in Figure 12 were loaded into memory and run through the impact detection algorithm. Note that this test uses pre-recorded impact data and was not directly carried out in the field. The goal of this implementation is simply to demonstrate the capability of deploying the developed impact detection system at the edge of wireless sensors. Future work will integrate this system with the Xnode and capture photos when an impact is detected.

**Figure 13:**
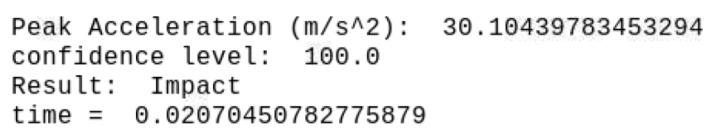
Results from edge impact detection.

Upon extracting features from the above signal, the features were used as input into the TensorFlow Lite model to run inference and get the predicted output. The model classified the signal as an impact event. In addition to the event classification, the model also outputs the peak acceleration by comparing accelerations in all three directions. Note that the peak acceleration is computed using both event and trigger accelerometer data. Furthermore, the confidence level of the detection is also shown, using the predicted probability output from the ANN model. Finally, using Python’s time module, the time for running the model on the event data was computed. The duration of the inference task was found to be 0.02 s. Additionally, the average inference time from five tests was found to be 0.0205 s, which signifies the potential to move towards a near-real-time impact detection system implemented at the edge of wireless sensors. Figure 13 shows the summary of the output from running the impact detection algorithm on the Raspberry Pi device.

## 5. Conclusions

In this paper, a robust impact detection system was developed using event-triggered wireless sensors and an artificial neural network. First, for the specific application of railroad bridge impact detection, a monitoring system was carefully selected. Subsequently, an event classification algorithm was employed to classify events occurring on railroad bridges and ultimately detect impacts. To validate the accuracy of the model, some of the data acquired were held out for testing, and a cross-validation approach was also employed. Additionally, a framework for edge classification of railroad bridge events was developed and demonstrated on the Raspberry Pi platform.

The results obtained showed an event classification accuracy of 98.67% and that the model was not overfit. Furthermore, an ROC AUC score of 0.99 shows the model is highly likely to positively identify an impact occurrence. The obtained F1 score of 0.98 demonstrates that the model is not expected to miss impact events when they occur as well as having a very low false positive rate. Finally, the developed system was implemented on the edge with a Raspberry Pi device, and the average inference time of 0.0205 s shows the potential of achieving near-real-time impact detection. This research and framework provide a foundation on which to build the Xnode edge deployment. Currently, the solution proposed in this work is limited to the specific bridges that have been instrumented. Future work includes the generalization of the prototype impact detection system developed in this paper. Likewise, the event classification algorithm framework can be increased by another layer to include the quantification and classification of the severity of detected impacts. Finally, an approach for a rapid condition assessment of bridges after impact events is under study.

## Figures and Tables

**Table 1:** Inputs for ANN model.

Feature	ADXL362 (Low-Fidelity)	LIS344ALH (High-Fidelity)
Maximum absolute acceleration	yes	yes
Dominant frequencies from FFT	no	yes
Center of mass	yes	no
Spectral energy	no	yes

**Table 2:** ANN model hyperparameters.

Parameter	Value
Loss function	Binary cross-entropy
Learning rate	0.001
Optimizer	Adam
No. of epochs	200

**Table 3:** Cross-validation evaluation scores.

Metric	Score
Mean accuracy	0.9867
ROC AUC	0.9900
F1	0.9793
Standard deviation	0.0267

## Data Availability

The data presented in this study are available on request from the corresponding author. The data are not publicly available due to privacy restrictions.
